# Microstructure-based hyperelastic models for closed-cell solids

**DOI:** 10.1098/rspa.2017.0036

**Published:** 2017-04-05

**Authors:** L. Angela Mihai, Hayley Wyatt, Alain Goriely

**Affiliations:** 1 School of Mathematics, Cardiff University, Senghennydd Road, Cardiff CF24 4AG, UK; 2 Mathematical Institute, University of Oxford, Woodstock Road, Oxford OX2 6GG, UK

**Keywords:** cellular solids, microstructural behaviour, constitutive responses, hyperelastic model, large strain deformation, finite-element simulation

## Abstract

For cellular bodies involving large elastic deformations, mesoscopic continuum models that take into account the interplay between the geometry and the microstructural responses of the constituents are developed, analysed and compared with finite-element simulations of cellular structures with different architecture. For these models, constitutive restrictions for the physical plausibility of the material responses are established, and global descriptors such as nonlinear elastic and shear moduli and Poisson’s ratio are obtained from the material characteristics of the constituents. Numerical results show that these models capture well the mechanical responses of finite-element simulations for three-dimensional periodic structures of neo-Hookean material with closed cells under large tension. In particular, the mesoscopic models predict the macroscopic stiffening of the structure when the stiffness of the cell-core increases.

## 1. Introduction

Cellular solids are the subject of intensive research efforts in biomedical applications, and many foams and sponges designed for cushioning and re-usability can be found in everyday life as well as in several industrial areas, e.g. microelectronics, aerospace and pharmaceutical processes [1–3]. The mechanics of cellular solids is also key in explaining the property or behaviour of fruit and legumes during storage or cooking, and are decisive for the perceived quality of food products [4–7]. Physical evidence suggests that the firmness of fruit decreases during preharvest ripening, which also involves a reduction in cellular pressure, and continues to decrease during post-harvest storage when the cell pressure further decreases [8]. Other mechanical and physiological factors, such as changes in cell size, wall thickness and composition, also contribute to changes in the macroscopic properties of fruit [9–13]. The relevant scale at which such phenomena occur, though beyond the capacity of the human eye, can be followed by mechanical analysis and mathematical models based on micro-structural evidence may be useful for the prediction of macroscopic behaviour [14–17].

The first microstructure-based model for a cellular solid is due to Gent & Thomas [18]. For this model, general isotropic linearly elastic open-cell foams subject to small strain deformations were assumed, and effective Young’s elastic modulus and the Poisson’s ratio were derived from the constitutive equations [19,20]. This model was extended to closed-cell foams containing an ideal gas by assuming that the elastic behaviour of the cell walls was essentially the same as for an open-cell foam of the same density, and adding contributions to the strain energy due to the enclosed gas phase and the surrounding atmosphere [21]. For cellular structures of nonlinearly elastic material under finite strain, a phenomenological continuum model is due to Blatz & Ko [22]. This model reduces to the Gent–Thomas model in the small strain limit [23,24]. The ellipticity of the Blatz–Ko model was analysed by Knowles & Sternberg [25]. In [26], it was noted that Hill’s energy functional of hyperelasticity [27] can be used to describe the simple special case of foams where the principal stresses are uncoupled, i.e. depend only upon the stretch ratio in the corresponding principal direction. These approaches are based on the Ogden-type strain energy function for incompressible materials [28] extended to the compressible case.

For open-cell solids with randomly oriented cell walls subject to large deformations, continuum isotropic hyperelastic models at the mesoscopic level have recently been obtained [29], provided that the cell walls are thin and subject to finite elastic stretches, and the wall joints were small and their deformation can be neglected.

In this study, the hyperelastic models for open-cell structures derived in [29] are extended and enhanced to account for the behaviour of cellular structures with closed cells (§2). For these structures, hyperelastic material components are considered satisfying the Baker–Ericksen (BE) inequalities stating that *the greater principal stress occurs in the direction of the greater principal stretch*, and of the pressure-compression (PC) inequalities stating that *each principal stress is a pressure* (*compression*) *or a tension according as the corresponding principal stretch is a contraction or an elongation* (*extension*) [30], pp. 155–159, and similar material responses are also found for the continuum models (§3). For these models, the nonlinear elastic and shear moduli and the Poisson’s ratio are obtained from the material characteristics of the constituents (§4). For numerical illustration, the mechanical performance of the mesoscopic models for structures with neo-Hookean components (§4e) is compared with finite-element simulations of three-dimensional structures with periodic, reproducible architecture (§5).

## 2. Hyperelastic models for structures with closed cells

We consider a cellular structure with closed cells subject to a triaxial stretch and denote by (**e**_1_,**e**_2_,**e**_3_) the usual orthonormal vectors for the Cartesian coordinates in the principal directions of the material deformation at the mesoscopic scale, and by {*α*_*i*_}_*i*=1,2,3_ the principal stretches, respectively. This assumption is similar to that for open-cells structures analysed in [29]. However, we note that, for the closed cells, the cell walls consist of both faces and edges unlike in the open-cells case where the cell walls contained only edges.

**Figure 1.**
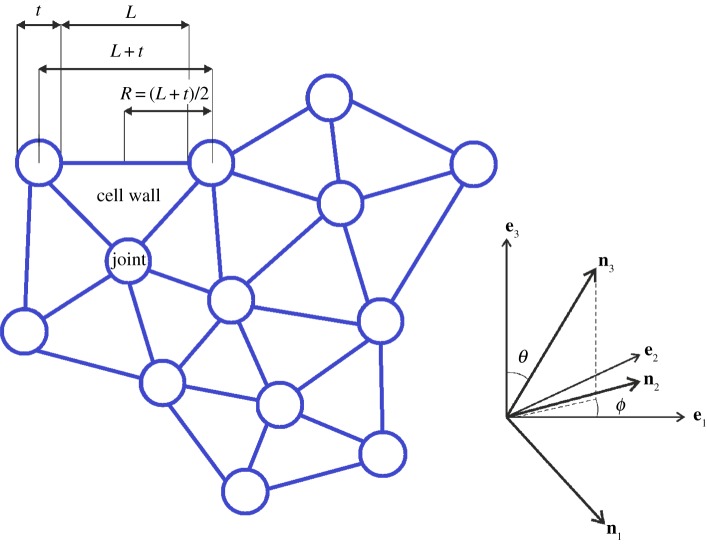
Schematic of cell walls and joints in a cellular structure, with the orthonormal vectors (**e**_1_,**e**_2_,**e**_3_) for the structure at the mesoscopic scale and (**n**_1_,**n**_2_,**n**_3_) for a cell wall. (Online version in colour.)

For this structure, we assume that, in the undeformed state, adjacent cell walls meet along cell edges of length *L*, and adjacent cell edges meet at spherical joints of width *t*, such that 0<*k*=*t*/*L*<1. We further assume that the deformation of the spherical joints is less significant than that of the cell walls and can be neglected (figure 1).

### (a) Cell walls under finite triaxial stretch

We define the displacement in the three-dimensional Euclidean space in the usual way:

2.1 u(X)=x−X,

where **X** are the Lagrangian (reference, material) and **x** are the Eulerian (current, spatial) coordinates, respectively. For a straight line joining two neighbouring particles with initial positions [**X**,**X**+*δ***X**], we assume that the particles are displaced to [**x**,**x**+*δ***x**], where by (2.1), **x**=**X**+**u**(**X**) and **x**+*δ***x**=**X**+*δ***X**+**u**(**X**+*δ***X**), respectively. By Taylor’s theorem,

2.2 $$\delta \text{x}=\delta \text{X}+\text{u}(\text{X}+\delta \text{X})-\text{u}(\text{X})=\delta \text{X}+(\delta \text{X}\cdot \nabla )\text{u}(\text{X})+O(|\delta \text{X}{|}^{2}),$$

where, using Einstein’s notation convention that repeated indices represents summation,

$$\delta \text{X}\cdot \nabla =\delta X_{k}\frac{\partial }{\partial X_{k}}.$$

For a cell edge subject to finite extension or compression in the direction situated along the vector *δ***X**, if *L*=|*δ***X**| and *l*=|*δ***x**| are the initial and the current lengths of the wall in this direction, respectively, then by (2.2),

2.3 $$l^{2}=|\delta \text{x}{|}^{2}\approx |\delta \text{X}+(\delta \text{X}\cdot \nabla )\text{u}(\text{X}){|}^{2},$$

and, using the summation convention:

2.4 $$l^{2}-L^{2}=2E_{ij}\delta X_{i}\delta X_{j},$$

where

2.5 $$\text{E}={\left(E_{ij}\right)}_{i,j=1,2,3},\;E_{ij}=\frac{1}{2}\left(\frac{\partial u_{i}}{\partial X_{j}}+\frac{\partial u_{j}}{\partial X_{i}}+\frac{\partial u_{k}}{\partial X_{i}}\frac{\partial u_{k}}{\partial X_{j}}\right)$$

is the Green–Lagrange strain tensor for the deforming edge.

If $\bar{L}=L+t=(1+k)L$ and $\bar{l}=l+t=l+kL$ are the lengths before and after the deformation, respectively, of a cell element comprising a cell wall bounded by cell edges and two half-joints at the end of each edge, then similarly to (2.4),

2.6 $${\bar{l}}^{2}-{\bar{L}}^{2}=2{\left(1+k\right)}^{2}{\bar{E}}_{ij}\delta X_{i}\delta X_{j},$$

where the Green–Lagrange strain tensor for the deforming cell element is defined as

2.7 $$\bar{\text{E}}={\left({\bar{E}}_{ij}\right)}_{i,j=1,2,3},\;{\bar{E}}_{ij}=\frac{{\left(\sqrt{2E_{ij}+\delta_{ij}}+k\delta_{ij}\right)}^{2}}{2{\left(1+k\right)}^{2}}-\frac{\delta_{ij}}{2},$$

with ***δ***=(*δ*_*ij*_)_*i*,*j*=1,2,3_ denoting the Kronecker delta.

For the cell wall and the cell element, the right Cauchy–Green tensors are, respectively,

2.8 $$\text{C}={\left(C_{ij}\right)}_{i,j=1,2,3},\;\text{C}=2\text{E}+\text{I}$$

and

2.9 $$\bar{\text{C}}={\left({\bar{C}}_{ij}\right)}_{i,j=1,2,3},\;\bar{\text{C}}=2\bar{\text{E}}+\text{I},$$

where **I** is the identity tensor.

Since $\text{C}=\mathrm{diag}(\lambda_{1}^{2},\lambda_{2}^{2},\lambda_{3}^{2})$ and $\bar{\text{C}}=\mathrm{diag}({\bar{\lambda }}_{1}^{2},{\bar{\lambda }}_{2}^{2},{\bar{\lambda }}_{3}^{2})$, where {*λ*_*i*_}_*i*=1,2,3_ and ${\left\{{\bar{\lambda }}_{i}\right\}}_{i=1,2,3}$ are the principal stretches for the cell wall and the cell element, respectively, by (2.7)–(2.9),

2.10 $$\lambda_{i}=(1+k){\bar{\lambda }}_{i}-k,\;i=1,2,3.$$

Let (**n**_1_,**n**_2_,**n**_3_) denote the orthonormal vectors for the Cartesian coordinates in the principal directions of the deforming cell wall, such that:

2.11 $$\begin{array}{rl} {\text{n}}_{1} & =-{\text{e}}_{1}\cos\theta \cos\phi -{\text{e}}_{2}\cos\theta \sin\phi +{\text{e}}_{3}\sin\theta ,\\ {\text{n}}_{2} & ={\text{e}}_{1}\sin\phi -{\text{e}}_{2}\cos\phi \\ \text{and}\;{\text{n}}_{3} & ={\text{e}}_{1}\sin\theta \cos\phi +{\text{e}}_{2}\sin\theta \sin\phi +{\text{e}}_{3}\cos\theta . \end{array}\}$$

Denoting by ${\text{C}}_{m}=\mathrm{diag}(\alpha_{1}^{2},\alpha_{2}^{2},\alpha_{3}^{2})$ the right Cauchy–Green tensor for the cellular solid at the mesoscopic level, we can write:

2.12 $$\begin{array}{rl} {\bar{\lambda }}_{1}^{2} & ={\text{n}}_{1}\cdot {\text{C}}_{m}{\text{n}}_{1}=\alpha_{1}^{2}\cos^{2}\theta \cos^{2}\phi +\alpha_{2}^{2}\cos^{2}\theta \sin^{2}\phi +\alpha_{3}^{2}\sin^{2}\theta ,\\ {\bar{\lambda }}_{2}^{2} & ={\text{n}}_{2}\cdot {\text{C}}_{m}{\text{n}}_{2}=\alpha_{1}^{2}\sin^{2}\phi +\alpha_{2}^{2}\cos^{2}\phi \\ \text{and}\;{\bar{\lambda }}_{3}^{2} & ={\text{n}}_{3}\cdot {\text{C}}_{m}{\text{n}}_{3}=\alpha_{1}^{2}\sin^{2}\theta \cos^{2}\phi +\alpha_{2}^{2}\sin^{2}\theta \sin^{2}\phi +\alpha_{3}^{2}\cos^{2}\theta . \end{array}\}$$

We assume that the cell walls are made from a homogeneous isotropic hyperelastic material described by a strain energy density function $W(I_{1},I_{2},I_{3})$, where *I*_1_, *I*_2_, *I*_3_ are the principal invariants of the Cauchy–Green tensor **C**:

2.13 $$I_{1}=\lambda_{1}^{2}+\lambda_{2}^{2}+\lambda_{3}^{2},\;I_{2}=\lambda_{1}^{2}\lambda_{2}^{2}+\lambda_{2}^{2}\lambda_{3}^{2}+\lambda_{3}^{2}\lambda_{1}^{2}\;\mathrm{and}\;I_{3}=\lambda_{1}^{2}\lambda_{2}^{2}\lambda_{3}^{2}.$$

For the deforming cell wall, the Cauchy stress tensor takes the form

2.14 $$\sigma =2J^{-1}\text{F}\frac{\partial W}{\partial \text{C}}{\text{F}}^{T},$$

where **F** is the deformation gradient and $J=\sqrt{I_{3}}$. When subject to a triaxial stretch, the non-zero components of the stress tensor are the diagonal ones:

2.15 σi=J−1λi∂W∂λi=J−1∂W∂(ln ⁡λi),i=1,2,3.

To ensure that the minimum strain energy is attained in the reference configuration, the relation

2.16 $$\frac{\partial W}{\partial \text{C}}=\text{0},$$

which corresponds to the unstressed state, must hold if *λ*_1_=*λ*_2_=*λ*_3_=1.

Defining the following principal invariants of the stretch tensor for the cell element:

2.17 $${\bar{i}}_{1}={\bar{\lambda }}_{1}+{\bar{\lambda }}_{2}+{\bar{\lambda }}_{3},\;{\bar{i}}_{2}={\bar{\lambda }}_{1}{\bar{\lambda }}_{2}+{\bar{\lambda }}_{2}{\bar{\lambda }}_{3}+{\bar{\lambda }}_{3}{\bar{\lambda }}_{1}\;\mathrm{and}\;{\bar{i}}_{3}={\bar{\lambda }}_{1}{\bar{\lambda }}_{2}{\bar{\lambda }}_{3},$$

we obtain:

2.18 $$\begin{array}{rl} I_{1} & ={\left[(1+k){\bar{i}}_{1}-3k\right]}^{2}-2[{\left(1+k\right)}^{2}{\bar{i}}_{2}-2k(1+k){\bar{i}}_{1}+3k^{2}],\\ I_{2} & ={\left[{\left(1+k\right)}^{2}{\bar{i}}_{2}-2k(1+k){\bar{i}}_{1}+3k^{2}\right]}^{2}\\ & \;-2[(1+k){\bar{i}}_{1}-3k][{\left(1+k\right)}^{3}{\bar{i}}_{3}-k{\left(1+k\right)}^{2}{\bar{i}}_{2}+k^{2}(1+k){\bar{i}}_{1}-k^{3}]\\ \text{and}\;I_{3} & ={\left[{\left(1+k\right)}^{3}{\bar{i}}_{3}-k{\left(1+k\right)}^{2}{\bar{i}}_{2}+k^{2}(1+k){\bar{i}}_{1}-k^{3}\right]}^{2}. \end{array}\}$$

Then the strain energy function describing the cell wall material can be written equivalently in terms of the invariants (2.17) as $W(I_{1},I_{2},I_{3})=\bar{W}({\bar{i}}_{1},{\bar{i}}_{2},{\bar{i}}_{3})$. We denote the principal invariants of the stretch tensor for the cellular solid at the mesoscopic level by:

2.19 $$i_{1}=\alpha_{1}+\alpha_{2}+\alpha_{3},\;i_{2}=\alpha_{1}\alpha_{2}+\alpha_{2}\alpha_{3}+\alpha_{3}\alpha_{1}\;\mathrm{and}\;i_{3}=\alpha_{1}\alpha_{2}\alpha_{3}.$$

Employing (2.12), we note that the principal invariants of the stretch tensor for the cell element are related to the principal invariants of the stretch tensor for the cellular solid by

2.20 $${\bar{i}}_{1}=i_{1},\;{\bar{i}}_{2}=i_{2}\;\mathrm{and}\;{\bar{i}}_{3}=i_{3}.$$

Therefore,

2.21 $$\bar{W}({\bar{i}}_{1},{\bar{i}}_{2},{\bar{i}}_{3})=\bar{W}(i_{1},i_{2},i_{3})$$

with no explicit dependence on the angles (*θ*,*ϕ*).

Then the strain energy per unit volume of the cellular solid at the mesoscopic level (designated by a subscript or superscript m) can be derived by taking the mean value of the cell wall energy W over the unit sphere, i.e.

2.22 $$\begin{array}{rl} W^{(m)} & =NV\frac{2}{\pi }\int_{0}^{\pi /2}\int_{0}^{\pi /2}\bar{W}(i_{1},i_{2},i_{3})\sin\theta \;d\theta \;d\phi \end{array}$$

2.23 $$\begin{array}{rl} & =\frac{NV}{2}\bar{W}(i_{1},i_{2},i_{3}), \end{array}$$

where *N* is the number of walls in a unit volume of cellular material and *V* is the volume of a cell wall.

The strain energy function (2.23) describes the behaviour of the deforming cellular solid at the mesoscopic scale, provided that the cells are empty and without internal pressure, and the cell walls are subject to finite triaxial stretch, without bending or buckling.

For the deforming cellular solid, the Cauchy stress tensor takes the form

2.24 $$\sigma^{(m)}=2J_{m}^{-1}{\text{F}}_{m}\frac{\partial W^{\left(m\right)}}{\partial {\text{C}}_{m}}{\text{F}}_{m}^{T},$$

where **F**_m_ is the deformation gradient and *J*_m_=*i*_3_. By (2.19) and (2.23), the principal components of the stress tensor (2.24) are

2.25 σi(m)=Jm−1αi∂W(m)∂αi=Jm−1∂W(m)∂(ln ⁡αi),i=1,2,3.

The minimum strain energy given by the relation

$$\frac{\partial W^{\left(m\right)}}{\partial {\text{C}}_{m}}=\text{0}$$

corresponds to the unstressed state, and by (2.25) and (2.16) it is attained if *α*_1_=*α*_2_=*α*_3_=1, i.e. when the cellular body is undeformed.

### (b) The volume fraction

To investigate the effect of the volume ratio between the elastic solid and the cellular material, we assume that *n* cell edges, each of undeformed length *L* and volume *V* , are meeting at a common joint of surface area

$$nA=4\pi {\left(\frac{t}{2}\right)}^{2}=\pi k^{2}L^{2},$$

where

$$k=\frac{t}{L}=\sqrt{\frac{nA}{\pi L^{2}}}.$$

Taking a representative volume of the cellular material at the mesoscopic scale in the undeformed state as a sphere of radius *R*=(*L*+*t*)/2=(1+*k*)*L*/2 centred at a joint, the volume of this sphere is equal to

2.26 $${\bar{V}}_{m}=\frac{4\pi R^{3}}{3}=\frac{\pi L^{3}{\left(1+k\right)}^{3}}{6}.$$

The volume of solid material contained in this sphere is

2.27 $$\bar{V}=\frac{nAL(1+k)}{2k}+\frac{4\pi {\left(t/2\right)}^{3}}{3}=\frac{\pi kL^{3}(1+k)}{2}+\frac{\pi k^{3}L^{3}}{6}=\frac{\pi L^{3}[3k(1+k)+k^{3}]}{6}.$$

By (2.26) and (2.27), the volume ratio is equal to

2.28 $$\rho_{m}=\frac{\bar{V}}{{\bar{V}}_{m}}=1-\frac{1}{{\left(1+k\right)}^{3}}$$

and increases as the parameter *k*∈(0,1) increases.

It follows that the corresponding volume fraction occupied by the cell interior is equal to 1−*ρ*_m_.

Without loss of generality, setting the unit sphere of cellular material at the mesoscopic scale as ${\bar{V}}_{m}=1$, by (2.26), it follows that

$$\frac{\pi L^{3}}{2}=\frac{3}{{\left(1+k\right)}^{3}},$$

which implies that the volume fraction of cell wall material in this sphere is equal to

2.29 $$NV=\frac{nAL}{2}=\frac{\pi kL^{3}(1+k)}{2}=\frac{3k}{{\left(1+k\right)}^{2}}.$$

Alternatively, setting the representative volume of the cellular body at the mesoscopic scale, in the undeformed state, as *n*_m_ spheres of radius *R*=(*L*+*t*)/2=(1+*k*)*L*/2, such that each sphere is centred at a joint, this volume is equal to $n_{m}{\bar{V}}_{m}$, where ${\bar{V}}_{m}$ is given by (2.26), and contains $n_{m}\bar{V}$ volume of solid material, with $\bar{V}$ defined by (2.27). Then the corresponding volume ratio

$$\rho_{m}=\frac{\left(n_{m}\bar{V}\right)}{\left(n_{m}{\bar{V}}_{m}\right)}=\frac{\bar{V}}{{\bar{V}}_{m}}=1-\frac{1}{{\left(1+k\right)}^{3}}$$

is the same as that given by (2.28).

In this case, taking the unit sphere of cellular material at the mesoscopic scale as $n_{m}{\bar{V}}_{m}=1$ implies

$$\frac{\pi L^{3}}{2}=\frac{3}{n_{m}{\left(1+k\right)}^{3}},$$

hence the associated volume fraction of cell wall material is equal to (2.29), i.e. depends only on the parameter *k*.

The same results are obtained if the unit volume is cubical in shape with the cuboid walls aligning with the cube edges and the cubical joints placed at the cube corners (see §5).

## 3. Material responses

In order that the behaviour of a hyperelastic material is physically realistic, there are some requirements taking the form of constraints on the constitutive equations, which are universally accepted.

For the cellular material with empty cells, by (2.20)–(2.21) and (2.23), the strain energy function $W^{\left(c\right)}(\alpha_{1},\alpha_{2},\alpha_{3})$ at the mesoscopic scale is independent of the orientation of the cell walls, and is a scalar multiple of the strain energy function for the cell element $\bar{W}({\bar{\lambda }}_{1},{\bar{\lambda }}_{2},{\bar{\lambda }}_{3})$. Hence, any constitutive constraints on the material responses at the mesoscopic are equivalent to similar constraints on the cell element.

Similarly, for the cellular material where the cells are filled with a hyperelastic material, since the strain energy function $W^{\left(f\right)}(\alpha_{1},\alpha_{2},\alpha_{3})$ given by (4.3) is a linear combination of the strain energy function for the cell element $\bar{W}(\alpha_{1},\alpha_{2},\alpha_{3})$ and the cell core $\tilde{W}(\alpha_{1},\alpha_{2},\alpha_{3})$, and is independent of the orientation of cells, any material constraints at the mesoscopic scale can be expressed equivalently as a linear combination of the corresponding constraints on the cell element and the cell core, respectively. Therefore, any constitutive constraints on the material responses at the mesoscopic scale can be expressed equivalently as a linear combination of the corresponding constraints on the cell element and the cell core, respectively.

In view of the above observations, here, we analyse in detail the case of empty cells, and indicate that the case of filled cells can be treated by analogy.

### (a) Baker–Ericksen inequalities

For a hyperelastic body subject to uniaxial tensile loading, the corresponding deformation is a simple extension in the direction of the tensile force, whereby the ratio between the tensile strain and the strain in the orthogonal direction is greater than one, if and only if the Baker–Ericksen (BE) inequalities stating that *the greater principal stress occurs in the direction of the greater principal stretch* hold [31,32].

For the cell wall material, the BE inequalities take the form [30, p. 158]

3.1 $$(\sigma_{i}-\sigma_{j})(\lambda_{i}-\lambda_{j})>0\;\text{if}\;\lambda_{i}\neq \lambda_{j},\;i,j=1,2,3,$$

where {*λ*_*i*_}_*i*=1,2,3_ and {*σ*_*i*_}_*i*=1,2,3_ are the principal stretches and the principal stresses, respectively.

For the cellular solid with empty cells, the principal Cauchy stress components take the following form in terms of the principal Cauchy stress components for the cell wall:

3.2 $$\sigma_{i}^{(m)}=(1+k)\frac{NV}{2}\frac{\lambda_{1}\lambda_{2}\lambda_{3}}{\alpha_{1}\alpha_{2}\alpha_{3}}\frac{\alpha_{i}}{\lambda_{i}}\sigma_{i}.$$

Hence, uniaxial tensile loading $\sigma_{3}^{\left(m\right)}=N^{\left(m\right)}>0=\sigma_{1}^{\left(m\right)}=\sigma_{2}^{\left(m\right)}$ for the cellular body implies uniaxial tensile loading for the cell wall, *σ*_3_=*N*>0=*σ*_1_=*σ*_2_. Assuming that the BE inequalities hold for the cell wall material, this implies that there is simple extension in the direction of the tensile force, i.e. *λ*_3_>*λ*_1_=*λ*_2_.

Taking *α*_*i*_=(*λ*_*i*_+*k*)/(1+*k*), *i*=1,2,3, for the cellular solid gives *α*_3_>*α*_1_=*α*_2_, i.e. uniaxial tensile loading produces simple extension. Hence, the BE inequalities also hold for the cellular solid, i.e.

3.3 $$(\sigma_{i}^{(m)}-\sigma_{j}^{\left(m\right)})(\alpha_{i}-\alpha_{j})>0\;\text{if}\;\alpha_{i}\neq \alpha_{j},\;i,j=1,2,3.$$

### (b) Pressure-compression inequalities

Another set of plausible constitutive constraints are the pressure-compression (PC) inequalities stating that *each principal stress is a pressure (compression) or a tension according as the corresponding principal stretch is a contraction or an elongation (extension)* [30, p. 155]. In practice, either or both of the mean versions of the PC conditions, which are physically more realistic, are acceptable,

3.4 $$\sigma_{1}(\lambda_{1}-1)+\sigma_{2}(\lambda_{2}-1)+\sigma_{3}(\lambda_{3}-1)>0$$

and

3.5 $$\sigma_{1}\left(1-\frac{1}{\lambda_{1}}\right)+\sigma_{2}\left(1-\frac{1}{\lambda_{2}}\right)+\sigma_{3}\left(1-\frac{1}{\lambda_{3}}\right)>0,$$

if not all *λ*_*i*_, *i*=1,2,3, are equal to 1.

Assuming that the inequalities (3.4)–(3.5) are satisfied for the cell wall material, and adding (3.4)–(3.5) multiplied by *k* gives

$$\begin{array}{rl} 0 & <\sigma_{1}(\lambda_{1}-1)+\sigma_{2}(\lambda_{2}-1)+\sigma_{3}(\lambda_{3}-1)\\ & \;+k\left[\sigma_{1}\left(1-\frac{1}{\lambda_{1}}\right)+\sigma_{2}\left(1-\frac{1}{\lambda_{2}}\right)+\sigma_{3}\left(1-\frac{1}{\lambda_{3}}\right)\right]\\ & =\left(1+\frac{k}{\lambda_{1}}\right)\sigma_{1}(\lambda_{1}-1)+\left(1+\frac{k}{\lambda_{2}}\right)\sigma_{2}(\lambda_{2}-1)+\left(1+\frac{k}{\lambda_{3}}\right)\sigma_{3}(\lambda_{3}-1). \end{array}$$

Taking *α*_*i*_=(*λ*_*i*_+*k*)/(1+*k*), *i*=1,2,3, for the cellular solid, by (3.2), it follows that

3.6 $$\sigma_{1}^{(m)}(\alpha_{1}-1)+\sigma_{2}^{\left(m\right)}(\alpha_{2}-1)+\sigma_{3}^{\left(m\right)}(\alpha_{3}-1)>0.$$

In this case also, (3.5) can be rewritten as

$$\left(1+\frac{k}{\lambda_{1}}\right)\sigma_{1}\left(1-\frac{1+k}{\lambda_{1}+k}\right)+\left(1+\frac{k}{\lambda_{2}}\right)\sigma_{2}\left(1-\frac{1+k}{\lambda_{1}+k}\right)+\left(1+\frac{k}{\lambda_{2}}\right)\sigma_{3}\left(1-\frac{1+k}{\lambda_{3}+k}\right)>0,$$

which is equivalent to

3.7 $$\sigma_{1}^{\left(m\right)}\left(1-\frac{1}{\alpha_{1}}\right)+\sigma_{2}^{\left(m\right)}\left(1-\frac{1}{\alpha_{2}}\right)+\sigma_{3}^{\left(m\right)}\left(1-\frac{1}{\alpha_{3}}\right)>0.$$

Hence, by (3.6)–(3.7), the PC inequalities hold for the cellular solid.

## 4. Cells filled with a hyperelastic core

When the cells are filled with a hyperelastic material, the contact forces acting between the cell core and the cell walls through the wall surface must also be taken into account at the mesoscopic level. Assuming that the cell core described by the strain energy function $\tilde{W}(i_{1},i_{2},i_{3})$, with the principal invariants of the stretch tensor for the cellular solid are given by (2.19), occupies the unit volume fraction 1−*ρ*_m_, with *ρ*_m_ given by (2.28), and is in active contact with the cell walls across the wall surface *Γ*_*c*_, with outer unit normal **N**, the elastic energy stored by the cellular body with filled cells (designated by a superscript f) under the triaxial stretch deformation is obtained as follows:

4.1 $$\begin{array}{rl} W^{\left(f\right)} & =W^{\left(m\right)}+\int_{\Gamma_{c}}\frac{\partial \tilde{W}}{\partial {\text{F}}_{m}}\text{N}\;dA \end{array}$$

4.2 $$\begin{array}{rl} & =\frac{NV}{2}\bar{W}(i_{1},i_{2},i_{3})+(1-\rho_{m})\frac{2}{\pi }\int_{0}^{\pi /2}\int_{0}^{\pi /2}\tilde{W}(i_{1},i_{2},i_{3})\sin\theta \;d\theta \;d\phi \end{array}$$

4.3 $$\begin{array}{rl} & =\frac{NV}{2}\bar{W}(i_{1},i_{2},i_{3})+\frac{1-\rho_{m}}{2}\tilde{W}(i_{1},i_{2},i_{3}), \end{array}$$

where **F**_m_ is the deformation gradient. The above formulation is based on the assumption that, in each cell, the core is a continuous material body occupying a compact domain of the three-dimensional Euclidean space, such that the interior of the body is an open, bounded, connected set, and a unit normal vector exists almost everywhere on its boundary [33,34].

Note that, in the particular case when, for the closed cells, NV is given by (2.29) and the cell core is made from the same material as the cell walls, i.e. $\tilde{W}(i_{1},i_{2},i_{3})=\bar{W}(i_{1},i_{2},i_{3})$, the strain energy (4.3) takes the form

4.4 $$\begin{array}{rl} W^{\left(f\right)} & =\frac{3k}{2{\left(1+k\right)}^{2}}\bar{W}(i_{1},i_{2},i_{3})+\frac{1}{2{\left(1+k\right)}^{3}}\bar{W}(i_{1},i_{2},i_{3}) \end{array}$$

4.5 $$\begin{array}{rl} & =\frac{3k^{2}}{2{\left(1+k\right)}^{3}}\bar{W}(i_{1},i_{2},i_{3})+\frac{1+3k}{2{\left(1+k\right)}^{3}}\bar{W}(i_{1},i_{2},i_{3}). \end{array}$$

Recalling that, for the mesoscopic model of open-cell structures in [29], NV=3*k*^2^/(1+*k*)^3^ and 1−*ρ*_m_=(1+3*k*)/(1+*k*)^3^, we can also interpret the first term in (4.5) as the mesoscopic energy for the open-cell structure and the second term as the energy of the material filling the space between the walls of the open cells.

### (a) Shear modulus

To compute the *nonlinear shear modulus* for the cellular solid with empty cells, we consider the simple shear deformation:

4.6 $$x_{1}=X_{1},\;x_{2}=X_{2}\;\mathrm{and}\;x_{3}=\gamma X_{1}+X_{3},$$

where *γ*>0 is constant. Then, the corresponding principal stretches satisfy:

$$\alpha_{1}^{2}=1+\frac{\gamma^{2}-\gamma \sqrt{\gamma^{2}+4}}{2}=\alpha^{-2},\;\alpha_{2}^{2}=1\;\mathrm{and}\;\alpha_{3}^{2}=1+\frac{\gamma^{2}+\gamma \sqrt{\gamma^{2}+4}}{2}=\alpha^{2}.$$

Noting that

$$\alpha =\frac{\gamma +\sqrt{\gamma^{2}+4}}{2}\;\text{and}\;\alpha^{-1}=-\frac{\gamma -\sqrt{\gamma^{2}+4}}{2},$$

we obtain the principal Cauchy stress components from (2.25). Then the *nonlinear shear modulus* [30, p. 175] for the cellular solid at the mesoscopic scale is defined as

4.7 $$\mu^{\left(m\right)}=\frac{\sigma_{3}^{\left(m\right)}-\sigma_{1}^{\left(m\right)}}{\alpha^{2}-\alpha^{-2}}.$$

In the limit of small shear strain, the *shear modulus* takes the form

4.8 $$\mu_{0}^{\left(m\right)}=\lim_{\gamma \to 0}\mu^{\left(m\right)}={\left(1+k\right)}^{2}\frac{NV}{2}\mu_{0},$$

where *μ*_0_ is the shear modulus for the cell wall material.

Similarly, for the cellular material with filled cells, since the strain energy function given by (4.3) is a linear combination of the strain energy function for the cell element and the cell core, the corresponding shear modulus at the mesoscopic scale is a linear combination of the corresponding parameters on the cell element and the cell core, respectively, i.e.

4.9 $$\mu_{0}^{\left(f\right)}={\left(1+k\right)}^{2}\frac{NV}{2}\mu_{0}+\frac{1-\rho_{m}}{2}{\tilde{\mu }}_{0},$$

where *μ*_0_ and ${\tilde{\mu }}_{0}$ are the shear moduli for the cell wall and the cell core, respectively.

When NV is given by (2.29), the shear modulus (4.9) takes the form

4.10 $$\mu_{0}^{\left(f\right)}=\frac{3k}{2}\mu_{0}+\frac{{\tilde{\mu }}_{0}}{2{\left(1+k\right)}^{3}}.$$

### (b) Elastic modulus

For the closed-cell material with empty cells, if uniaxial loading causes a simple tension or compression in the direction of the tensile force with *α*_1_=*α*_2_<*α*_3_, such that $\sigma_{1}^{(m)}=\sigma_{2}^{\left(m\right)}=0$ and $\sigma_{3}^{(m)}=N^{\left(m\right)}$, then the *nonlinear elastic modulus* is defined as the slope of curve representing the axial stress versus the associated logarithmic strain, i.e.

4.11 E(m)=∂N(m)∂(ln ⁡α3).

In the linear elastic limit, the Young’s modulus for the cellular material with empty cells is equal to

4.12 $$E_{0}^{\left(m\right)}=\lim_{\alpha_{3}\to 1}E^{\left(m\right)}={\left(1+k\right)}^{2}\frac{NV}{2}E_{0},$$

where *E*_0_ is the Young’s modulus for the cell-wall material.

For the closed-cell material with filled cells, by analogous calculations, the Young’s modulus at the mesoscopic scale is a linear combination of the corresponding parameters for the cell wall and the cell core, respectively, i.e.

4.13 $$E_{0}^{\left(f\right)}={\left(1+k\right)}^{2}\frac{NV}{2}E_{0}+\frac{1-\rho_{m}}{2}{\tilde{E}}_{0},$$

where *E*_0_ and ${\tilde{E}}_{0}$ are the Young’s moduli for the cell wall and the cell core, respectively.

When NV is defined by (2.29), the Young’s modulus (4.13) takes the form

4.14 $$E_{0}^{\left(f\right)}=\frac{3k}{2}E_{0}+\frac{{\tilde{E}}_{0}}{2{\left(1+k\right)}^{3}}.$$

### (c) Poisson’s ratio

For an elastic material, if uniaxial loading causes a simple tension or compression in the direction of the tensile force, then the *nonlinear Poisson’s ratio* can be computed as the negative quotient of the logarithmic strain in an orthogonal direction to that of the logarithmic strain in the third direction [23]. For the cell wall and the cellular body with closed cells, respectively, this Poisson’s function is

4.15 ν=−ln ⁡λ1ln ⁡λ3,ν(m)=−ln ⁡α1ln ⁡α3.

Then, in the linear elastic limit *λ*_3_→1, the resulting Poisson’s ratio is

4.16 $$\nu_{0}^{(m)}=\lim_{\alpha_{3}\to 1}\frac{1-\alpha_{1}}{\alpha_{3}-1}=\lim_{\lambda_{3}\to 1}\frac{1-\lambda_{3}^{-\nu }}{\lambda_{3}-1}=\nu_{0},$$

where *ν*_0_ is the Poisson’s ratio for the cell wall material.

When the cells are filled with an elastic core that is softer than the cell walls, the Poisson’s ratio at the mesoscopic scale is equal to

4.17 $$\nu_{0}^{\left(f\right)}=\frac{k}{1+k}\nu_{0}+\frac{1}{1+k}{\tilde{\nu }}_{0},$$

where *ν*_0_ and ${\tilde{\nu }}_{0}$ are the Poisson’s ratios for the cell wall and the cell core, respectively.

### (d) The cell-size effect

In order to capture the independent influence of the cells number on the elastic behaviour of a cellular body under large deformations characteristic to some cellular structures, namely that, for structures made from the same volume of hyperelastic material, the stiffness increases as the number of cells increases while the ratio between the thickness and the length of the walls remains fixed [35,7,36,29], we replace the strain energy function (4.3) with

4.18 $$W^{\left(cf\right)}=\eta W^{\left(f\right)},$$

where *η*>0 is chosen so that, for structures containing the same volume of cell wall material and having similar cell geometries, if the number of walls in a unit volume of cellular material *N* and the volume of a cell wall *V* remain unchanged, then *η* increases as the number of cells increases.

For the hyperelastic material described by (4.18), the nonlinear shear and elastic moduli take the form, respectively,

4.19 $$\mu^{\left(cf\right)}=\eta \mu^{\left(f\right)}$$

and

4.20 $$E^{\left(cf\right)}=\eta E^{\left(f\right)}.$$

In practice, for structures under tension in the third direction, the weight *η* can be chosen so that the effective elastic modulus for the continuum model is equal to

4.21 $$E_{\mathrm{eff}}=\lim_{\alpha_{3}\to 1}{\bar{E}}_{\mathrm{eff}},$$

where ${\bar{E}}_{\mathrm{eff}}$ is the slope of the effective Cauchy stress *vs.* the effective logarithmic strain curve for the closed-cell structure. Recall that the effective value of a symmetric tensor **s** [37,38] is defined as

4.22 $$\begin{array}{rl} s_{\mathrm{eff}} & =\sqrt{\frac{3}{2}[(\text{s}-\frac{1}{3}\;\text{tr}(\text{s})\text{I}):(\text{s}-\frac{1}{3}\;\text{tr}(\text{s})\text{I})]}\\ & =\sqrt{s_{11}^{2}+s_{22}^{2}+s_{33}^{2}-s_{11}s_{22}-s_{22}s_{33}-s_{33}s_{11}+3(s_{12}^{2}+s_{13}^{2}+s_{23}^{2})}. \end{array}$$

Then

4.23 $$\eta =\frac{2E_{\mathrm{eff}}}{{\left(1+k\right)}^{2}NVE_{0}+(1-\rho_{m}){\tilde{E}}_{0}}.$$

### (e) Mesoscopic model for closed-cells of neo-Hookean material

We now specialise our model to the case where the cell wall and the cell core materials are described by the compressible neo-Hookean models

4.24 W(I1,I2,I3)=μ2(I1−3−ln ⁡I3)+λ2(ln ⁡I31/2)2,

and

4.25 W~(I1,I2,I3)=μ~2(I1−3−ln ⁡I3)+λ~2(ln ⁡I31/2)2,

where *μ*>0, *λ*>0 and $\tilde{\mu }>$, $\tilde{\lambda }>0$ are constant parameters, such that $\mu >\tilde{\mu }$, i.e. the walls are stiffer than the core.

For the cell wall and the cell core, the principal Cauchy stress components are, respectively,

4.26 $$\sigma_{i}=\frac{1}{\lambda_{1}\lambda_{2}\lambda_{3}}[\mu (\lambda_{i}^{2}-1)+\lambda \ln(\lambda_{1}\lambda_{2}\lambda_{3})],\;i=1,2,3,$$

and

4.27 $${\tilde{\sigma }}_{i}=\frac{1}{\lambda_{1}\lambda_{2}\lambda_{3}}[\tilde{\mu }(\lambda_{i}^{2}-1)+\tilde{\lambda }\ln(\lambda_{1}\lambda_{2}\lambda_{3})],\;i=1,2,3,$$

In this case, the corresponding BE inequalities (3.1) are equivalent to *μ*>0 and $\tilde{\mu }>0$.

In this case, the strain energy function at the mesoscopic scale (4.18) is

4.28 W(cf)(i1,i2,i3)=W(c)(i1,i2,i3)+η1−ρm2[μ~2(i12−2i2−3−ln ⁡i32)+λ~2(ln ⁡i3)2]=ηNV2μ2[(1+k)2(i12−2i2)−2k(1+k)i1−3(1−k2)]−η(1+k)NV2μln ⁡[(1+k)3i3−k(1+k)2i2+k2(1+k)i1−k3]+ηNV2λ2{ln ⁡[(1+k)3i3−k(1+k)2i2+k2(1+k)i1−k3]}2+η1−ρm2[μ~2(i12−2i2−3−ln ⁡i32)+λ~2(ln ⁡i3)2].

The corresponding principal Cauchy stress components are

4.29 σi(cf)=η(1+k)NV2μαiα1α2α3[αi(1+k)−k−1αi(1+k)−k]+η(1+k)NV2λαiα1α2α3ln ⁡[(1+k)3i3−k(1+k)2i2+k2(1+k)i1−k3]αi(1+k)−k+η1−ρm21α1α2α3[μ~(αi2−1)+λ~ln ⁡i3],i=1,2,3.

In the small strain limit, the shear modulus is equal to

4.30 $$\mu_{0}^{\left(cf\right)}=\eta {\left(1+k\right)}^{2}\frac{NV}{2}\mu +\eta \frac{1-\rho_{m}}{2}\tilde{\mu },$$

the corresponding Young’s modulus takes the form

4.31 $$E_{0}^{\left(cf\right)}=\eta {\left(1+k\right)}^{2}\frac{NV}{2}E+\eta \frac{1-\rho_{m}}{2}\tilde{E},$$

where

4.32 $$E=\mu \frac{2\mu +3\lambda }{\mu +\lambda },\;\tilde{E}=\tilde{\mu }\frac{2\tilde{\mu }+3\tilde{\lambda }}{\tilde{\mu }+\tilde{\lambda }},$$

and the Poisson’s ratio is

4.33 $$\nu_{0}^{\left(cf\right)}=\frac{k}{1+k}\frac{\lambda }{2(\lambda +\mu )}+\frac{1}{1+k}\frac{\tilde{\lambda }}{2(\tilde{\lambda }+\tilde{\mu })}=\frac{k}{1+k}\nu +\frac{1}{1+k}\tilde{\nu }.$$

For the cellular solid with empty cells, the strain energy function is $W^{\left(c\right)}(i_{1},i_{2},i_{3})$ and the corresponding parameters are obtained by setting *ρ*_m_=1 in the derivation of the parameters for the case with filled cells.

## 5. Numerical examples

In this section, the mechanical performance of the continuum hyperelastic models is compared numerically to that of finite-element simulations of three-dimensional periodic closed-cell structures under large tension. The finite-element models were created in SolidWorks and imported into the Finite Elements for Biomechanics (FEBio) software [39], where a mesh refinement study was performed. The undeformed structures created in SolidWorks are shown as figures 4 and 5 in the Appendix.

Every structure is deformed by imposing the following boundary conditions: the lower external horizontal face is fixed in the *Y* /second/vertical-direction and free to slide in the *X*/first/horizontal-direction and in the *Z*/third/out-of-plane-direction; the upper external horizontal face is under prescribed tension in the *Y* -direction and is free to slide in the *X* and *Z*-directions; the remaining external and internal cell faces deform freely.

For each structure, the cell walls form a continuous piece of solid material (see appendix), described by the compressible neo-Hookean model (4.24) with *μ*=*E*/[2(1+*ν*)] and *λ*=*νE*/[(1+*ν*)(1−2*ν*)], where *E*=0.1 MPa and *ν*=0.49. When the cells are filled, the cell core is characterized by the neo-Hookean model (4.25) with $\tilde{\mu }=\tilde{E}/[2(1+\tilde{\nu })]$ and $\tilde{\lambda }=\tilde{\nu }\tilde{E}/[(1+\tilde{\nu })(1-2\tilde{\nu })]$, where, (*I*) $\tilde{E}=0.01\;\mathrm{MPa}$ and $\tilde{\nu }=0.49$ for structure 1 and model 1, i.e. the cell core is 10 times softer than the cell walls; (*II*) $\tilde{E}=0.005\;\mathrm{MPa}$ and $\tilde{\nu }=0.49$ for structure 2 and model 2, i.e. the cell core is 20 times softer than the cell walls; and (*III*) $\tilde{E}=0.001\;\mathrm{MPa}$ and $\tilde{\nu }=0.49$ for structure 3 and model 3, i.e. the cell core is 100 times softer than the cell walls.

The corresponding effective modulus *E*_eff_ is computed as the slope of the mean effective Cauchy stress (normalized by *E*=0.1 MPa) *vs.* the mean effective logarithmic strain curve, and is shown up to 20% vertical tension, together with the effective modulus for the associated continuum model. The mean value was calculated as the sum of the values on all the finite elements divided by the number of elements.

### (a) Closed cubical cells

If the unit volume is cubical in shape, then six cell edges, each of undeformed length *L*, are meeting at a common joint of surface area 6*t*^2^=6*k*^2^*L*^2^, where *k*=*t*/*L*. Taking a representative volume of the cellular material scale in the undeformed state as a cube of side *L*+*t*=*L*(1+*k*), the volume of this cube is ${\bar{V}}_{m}={\left(L+t\right)}^{3}=L^{3}{\left(1+k\right)}^{3}$, while the volume of solid material contained in this cube is $\bar{V}=L^{3}[3k(1+k)+k^{3}]$. Hence the volume ratio is

$$\frac{\bar{V}}{{\bar{V}}_{m}}=1-\frac{1}{{\left(1+k\right)}^{3}}$$

and is equal to (2.28). Setting ${\bar{V}}_{m}=1$, the volume fraction of cell wall material in this cube is

$$NV=\frac{6t^{2}L(1+k)}{2k}=\frac{3k}{{\left(1+k\right)}^{2}},$$

and is equal to (2.29).

### (b) Closed hexagonal prismatic cells

When the unit volume is an hexagonal prism, five edges, each of undeformed length *L*, are meeting at a common joint of surface area 5*t*^2^=5*k*^2^*L*^2^, where *k*=*t*/*L*. In this case, the volume fraction of cell wall material is is equal to two-thirds of the volume fraction (2.29), i.e.

$$NV=\frac{5t^{2}L(1+k)}{2k}=\frac{5k}{2{\left(1+k\right)}^{2}}.$$

**Figure 2.**
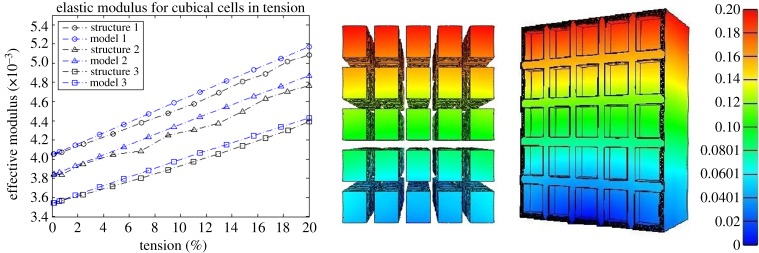
A comparison between model (continuous line) and simulation (dashed line) of the effective elastic modulus. The cell-core is 10 times softer than the cell walls for structure 1, 20 times softer than the cell walls for structure 2, and 100 times softer than the walls for structure 3. The closed cubical cells and their inclusions are shown at 20% (right) tension in the vertical direction (colour bar showing the displacement in the same direction). (Online version in colour.)

**Figure 3.**
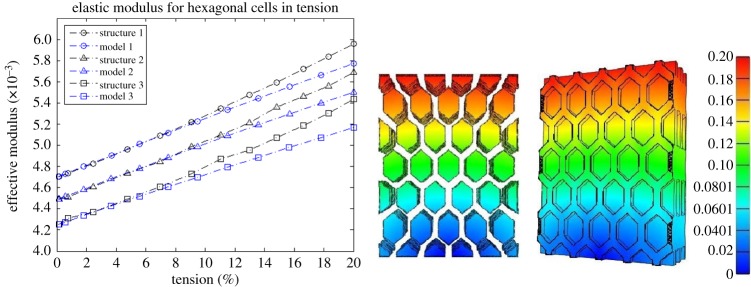
A comparison between model (continuous line) and simulation (dashed line) of the effective elastic modulus. The cell-core is 10 times softer than the cell walls for structure 1, 20 times softer than the cell walls for structure 2, and 100 times softer than the walls for structure 3. The closed hexagonal prismatic cells and their inclusions are shown at 20% (right) tension in the vertical direction (colour bar showing the displacement in the same direction) is shown. (Online version in colour.)

**Figure 4.**
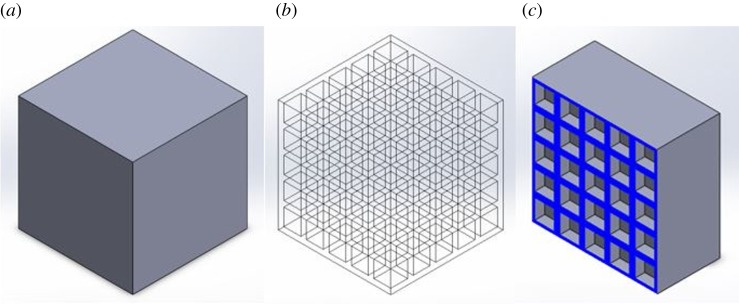
Closed-cell structure with 5×5×5 cubical cells, showing the undeformed structure (*a*), the distribution of cells (*b*) and a plane cross section parallel to some of the cell walls (*c*). (Online version in colour.)

**Figure 5.**
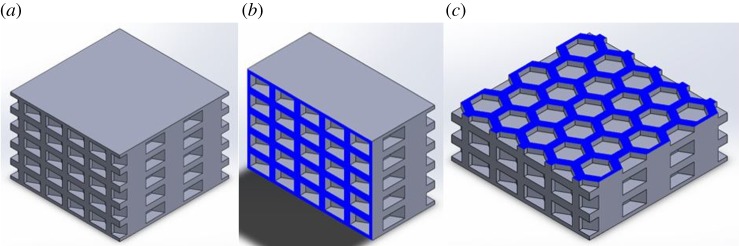
Closed-cell structure with hexagonal prismatic cells, showing the undeformed structure (*a*), and plane cross sections parallel to some of the cell walls (*b*) and (*c*). (Online version in colour.)

For the numerical examples involving three-dimensional periodic structures with cubical and hexagonal prismatic cells, respectively, the numerical results plotted in figures 2 and 3 show that, if the deformation of the cell walls is close to the triaxial stretch assumed theoretically, then the continuum models offer a good approximation for those structures. These models also correctly predict the macroscopic stiffening of the structure as the stiffness of the cell core increases.

## 6. Conclusion

Bridging the microstructural responses of individual cells with the apparent macrostructural behaviour is a challenging modelling task in materials science, as far as soft cellular structures with components exhibiting material nonlinear elasticity are concerned. To date, there is no established continuum model for this type of structures, even though, in principle, this should stand on the shoulders of the existing nonlinear elasticity theory. Here, we strived to make our models analytically tractable, and in addition, demonstrate their numerical performance through suitable comparisons with finite-element models of cellular structures under similar loading conditions.

Theoretically, we devised a class of mesoscale hyperelastic models applicable to closed cellular structures with randomly oriented, isotropic hyperelastic cell walls, whereby the elastic behaviour at the cell level is reflected at the continuum mesoscale level. To validate our theoretical results, computationally, for the finite-element models, the structural geometry and the cell-wall material need to be specified, and we chose periodic cellular structures of neo-Hookean material for exemplification. When tested computationally, our continuum models show both the expected qualitative behaviour predicted theoretically and very good numerical agreement with the finite-element models of various cellular structures under similar loads. In particular, our models capture the macroscopic stiffening of a structure when the stiffness of the cell core increases.

The hyperelastic models that we developed are suitable for incorporation in an adaptive multi-scale approach for the finite-element analysis of cellular bodies, whereby a cellular structure is first represented as a continuum hyperelastic material, then the areas where the stress field is in equilibrium with the load distribution and reaches critical values are re-modelled at the cellular level to capture the local mechanical effects.

## Supplementary Material

Preview-type source file of FEBios simulation for cellular structure with closed cubic cells of neo-Hookean material.

## Supplementary Material

Preview-type source file of FEBio simulation for cellular structure with closed hexagonal prismatic cells of neo-Hookean material.

## Data accessibility

The datasets supporting this article have been uploaded as electronic supplementary material.

## Appendix A. Structural geometries for computer simulations

In this appendix, we show the undeformed closed-cell structures created in SolidWorks which have been analysed in §5 (figures 4 and 5).
